# Evolving concepts in bone infection: redefining “biofilm”, “acute vs. chronic osteomyelitis”, “the immune proteome” and “local antibiotic therapy”

**DOI:** 10.1038/s41413-019-0061-z

**Published:** 2019-07-15

**Authors:** Elysia A. Masters, Ryan P. Trombetta, Karen L. de Mesy Bentley, Brendan F Boyce, Ann Lindley Gill, Steven R. Gill, Kohei Nishitani, Masahiro Ishikawa, Yugo Morita, Hiromu Ito, Sheila N. Bello-Irizarry, Mark Ninomiya, James D. Brodell, Charles C. Lee, Stephanie P. Hao, Irvin Oh, Chao Xie, Hani A. Awad, John L. Daiss, John R. Owen, Stephen L. Kates, Edward M. Schwarz, Gowrishankar Muthukrishnan

**Affiliations:** 1 Center for Musculoskeletal Research, University of Rochester Medical Center, Rochester, NY USA; 2 Department of Biomedical Engineering, University of Rochester Medical Center, Rochester, NY USA; 3 Department of Pathology and Laboratory Medicine, University of Rochester Medical Center, Rochester, NY USA; 4 Department of Orthopaedics, University of Rochester Medical Center, Rochester, NY USA; 5 Department of Microbiology & Immunology, University of Rochester Medical Center, Rochester, NY USA; 6 Department of Orthopaedic Surgery, Kyoto University, Kyoto, Japan; 7 Department of Orthopaedic Surgery, Virginia Commonwealth University Medical Center, Richmond, VA USA

**Keywords:** Bone, Diseases

## Abstract

Osteomyelitis is a devastating disease caused by microbial infection of bone. While the frequency of infection following elective orthopedic surgery is low, rates of reinfection are disturbingly high. *Staphylococcus aureus* is responsible for the majority of chronic osteomyelitis cases and is often considered to be incurable due to bacterial persistence deep within bone. Unfortunately, there is no consensus on clinical classifications of osteomyelitis and the ensuing treatment algorithm. Given the high patient morbidity, mortality, and economic burden caused by osteomyelitis, it is important to elucidate mechanisms of bone infection to inform novel strategies for prevention and curative treatment. Recent discoveries in this field have identified three distinct reservoirs of bacterial biofilm including: *Staphylococcal* abscess communities in the local soft tissue and bone marrow, glycocalyx formation on implant hardware and necrotic tissue, and colonization of the osteocyte-lacuno canalicular network (OLCN) of cortical bone. In contrast, *S. aureus* intracellular persistence in bone cells has not been substantiated in vivo, which challenges this mode of chronic osteomyelitis. There have also been major advances in our understanding of the immune proteome against *S. aureus*, from clinical studies of serum antibodies and media enriched for newly synthesized antibodies (MENSA), which may provide new opportunities for osteomyelitis diagnosis, prognosis, and vaccine development. Finally, novel therapies such as antimicrobial implant coatings and antibiotic impregnated 3D-printed scaffolds represent promising strategies for preventing and managing this devastating disease. Here, we review these recent advances and highlight translational opportunities towards a cure.

## Introduction

Osteomyelitis, defined as inflammation of the bone often caused by bacterial infection, is one of the oldest diseases in history.^1^ Infection of bone can be caused by *endogenous* seeding, known as hematogenous osteomyelitis,^2^ or by *exogenous* seeding, via contamination of a fracture site or surgical hardware during implantation.

With over 1.5 million total hip and total knee replacement (TKR) procedures performed each year,^3,4^ bone infection remains the most severe and devastating risk associated with orthopedic implants. It has been understood for decades that the addition of a foreign material to a biological environment provides a haven for bacterial attachment and colonization.^5–8^ Additionally, movement-induced wear on orthopedic prostheses causes the release of debris, resulting in local inflammation, and creating a favorable site for the development of infection.^9^

While advances in prophylaxis and aseptic surgical technique have decreased the incidence of orthopedic infection following hip or knee arthroplasty, rigorous intervention studies (e.g. outcomes from the Surgical Care Improvement Project (SCIP)^10^) have demonstrated that infection rates for elective surgery cannot be reduced below 1%–2%.^10–13^ Additionally, rates of recurrent or persistent infection following a two-stage revision surgery are still as high as 33%.^13–15^ Despite infection treatment strategies such as surgical site debridement, complete hardware exchange, and aggressive long-term antimicrobial therapy, infections continue to recur. In total, the cost for treatment of implant-associated osteomyelitis is projected to exceed $1.62 billion by 2020.^16^ These data are consistent with the conclusions from the 2018 International Consensus Meeting on Musculoskeletal Infection, which found that the incidences of infection for all orthopedic subspecialties range from 0.1% to 30%, at a cost of $17 000–$150 000 per patient.^13^

An astounding 75% of osteomyelitis cases are caused by pathogens of the *Staphylococcus* genus.^17,18^ Specifically, *Staphylococcus aureus* is the most common pathogen isolated from implant-associated ostemyelitis^17,19,20^ and over 50% of cases are caused by hard-to-treat methicillin-resistant *S. aureus* (MRSA) strains.^21^ For these reasons, *S. aureus* will be the primary focus of this review. Other osteomyelitis-causing pathogens include *Enterococcus, Pseudomonas*, and *Streptococcus* species.^17^

*S. aureus* is an extremely versatile opportunistic pathogen that can infect nearly every organ system in the human body causing life-threatening disease,^22^ while maintaining the ability to asymptomatically colonize 20%–60% of individuals.^23^ The invasive success of *S. aureus* infection can be attributed to its arsenal of virulence factors and resistance mechanisms including secreted toxins,^24^ adherence as a means of immune evasion,^25^ biofilm formation,^26,27^ the creation of slow growing small colony variant (SCV) subpopulations,^28,29^ and the development of antimicrobial resistance.^30^ As a result of these highly evolved pathogenic mechanisms of persistence, clinical *S. aureus* osteomyelitis recurrence after decades of quiescence remains an important problem.^31–33^

It has been over 200 years since Sir Benjamin Brodie described the bacterial abscess in bone that bears his name,^34^ and 40 years since William Costerton’s biofilm hypothesis explained the pathogenic mode of existence by which sessile bacteria adhere to implants and necrotic tissue during chronic infection.^35^ Based on these fundamental concepts of bone infection, a standard of care treatment for implant-associated osteomyelitis, most notably prosthetic joint infection (PJI), was established in the 1970s and involves: (1) removal of the infected implant, (2) extensive surgical debridement of adjacent bone and soft tissues, and (3) filling of the bone void with antibiotic-loaded acrylic cement. In a seminal, retrospective analysis of 825 one-stage reimplantations using this approach for infected total hip arthroplasties, Buchholz et al. documented in 1984 that *S. aureus* was the most commonly encountered organism, and that the 5-year success (survival) rate was only 77%.^36^ Remarkably, the results from the 2018 International Consensus Meeting on Musculoskeletal Infections reported no changes in PJI infection rates, the primary pathogen, treatment algorithm, and poor outcomes, since this original standard of care was established half a century ago.^8,13,37^ However, there have been recent basic and translational science advances in our understanding of microbial pathogenesis, antibiotic resistance, and the osteoimmunology of bone infection that warrant reevaluation of clinical management for bone infection. Thus, the goal of this review is to highlight these potential breakthroughs, which challenge the scientific premise of established paradigms, including “acute and chronic” osteomyelitis, intracellular infection of bone cells, and the efficacy of antibiotic-laden bone cement. Additionally, by reviewing emerging concepts in bone infection, with specific focus on *S. aureus* pathogenesis in chronic osteomyelitis, we aim to discuss novel diagnostics, immunizations, and therapies that could be transformative for this harmful condition.

## Treatment of peri-PJI

Although the rate of infection in primary hip and knee arthroplasties is low, rates of reinfection are reported as high as 40%.^13,15^ These infections frequently result in implant failure due to devastating effects on bone and soft tissue, requiring hardware removal for successful treatment. Because of the absence of an effective therapy for implant-associated osteomyelitis and lack of consistent guidelines for treatment, there is great debate over the appropriate procedure for managing total joint replacement (TJR) infections.

Principles of curative surgery in implant-associated osteomyelitis involve a variety of factors including infection severity, the infecting pathogen, and the condition of the patient.^38^ In the case of “acute” or low-level infection without complicating factors, such as significant comorbidity or loosening of the prosthesis, a surgeon may choose to treat with a regimen of debridement, antibiotics, irrigation, and retention (DAIR).^39–41^ Historically, DAIR has shown success rates as low as 14%,^42^ and as high as 100%.^43^ More recent studies have found DAIR success rates in appropriate patients to be ~70%–75%.^40,44–47^ Not surprisingly, retrospective studies of PJI revealed that patient health and the species of infecting organism significantly affected the success of DAIR treatment.^48^ DAIR failures and implant-associated bone infections that are not in this “acute” category must undergo hardware removal-revision surgery. Unfortunately, specific guidelines for the choice of treatment with hardware retention are widely variable and lacking scientific evidence,^49–52^ making the important decision to retain hardware a contentious and highly subjective choice.

As infection progresses, eradication of bacteria from implant hardware surfaces becomes the primary concern of surgeons because of the formation of robust biofilms. Therefore, a complete hardware exchange is recommended in cases of “chronic” or longer duration infection. Hardware exchange surgeries can be performed in 1 or 2 stages, depending on the use of local antibiotic delivery mechanisms, such as antibiotic-laden beads or bone cement that must be removed in a follow-up revision surgery. The advantage of single-stage vs. two-stage is currently unclear and seemingly largely dependent on the specific case.^53,54^

**Fig. 1:**
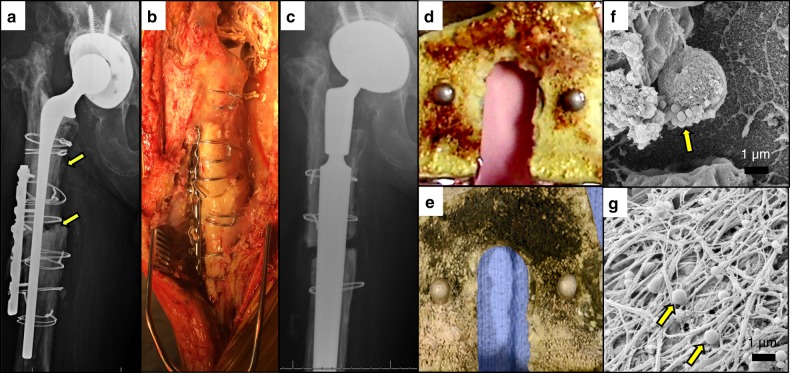
Removal of necrotic bone and biofilm contaminated components during revision surgery for MRSA-infected total joint replacements (TJR). **a**–**c** The indications for this single-stage revision for a MRSA-infected total hip replacement is shown. **a** Radiographic evidence of the septic TJR in the pre-op X-ray are periosteal reaction and a non-united femoral fracture (yellow arrows). **b** The open infected thigh requires removal of necrotic soft tissue and white (dead) bone, adjacent to live (red) bone that needs to be retained for successful limb salvage. Complete removal of the dead bone, cement, and necrotic tissues creates a healthier environment for the new prosthesis. **c** Post-op X-ray of the femoral defect with modular hip prosthesis. **d**–**g** Bacterial biofilm on explanted hardware components. Photographs of the surface of a femoral total knee replacement component before (**d**) and after (**e**) osmium tetroxide staining identifying bacterial biofilm on the bone cement. **f** SEM of the explanted hardware reveals biofilm bacteria (yellow arrow) on the surface of the implant (x10 000) and **g** bacteria attached to fibrin on the explanted hardware (x10 000)

Initially, progressive osteomyelitis is evaluated by radiology to visualize bone lysis or erosion with associated soft tissue pathology (Fig. 1a). Single-stage revision is typically pursued if the microorganism is treatable, if the bone remains stable when the hardware is removed,^55^ or if the patient’s health suggests the need to limit surgical procedures. In a single-stage revision surgery, hardware removal and replacement are conducted in a single surgical procedure along with radical debridement of infected tissue and antibiotic treatment (Fig. 1b, c). When infected hardware is removed following revision surgery, extensive biofilm can be visualized on the surface of the implants immediately adjacent to bone (Fig. 1d, e). High magnification scanning electron micrographs show *S. aureus* embedded within an extracellular matrix of biofilm (Fig. 1e, g).

On the other hand, in a two-stage revision procedure the hardware is removed. Antibiotic-laden bone cement (poly(methyl methacrylate (PMMA)) beads or spacers are implanted for dead space management and to locally deliver a high concentration of antibiotics, such as gentamicin or vancomycin^56^ to reduce detrimental systemic effects. While local delivery of antibiotics can be effective in eradicating infection, it may also introduce additional risks leading to bacterial resistance and recurrence of infection. It has been shown that antibiotic release efficiency from beads is low due to an early burst release, resulting in antibiotic concentrations below the minimum inhibitory concentrations.^57^ Administration of antibiotics in low concentrations can trigger the formation of antibiotic-resistant pathogens and SCVs, leading to recurrence of infection.^29^ Additionally, beads and bone cement spacers that are no longer eluting antibiotics provide additional surfaces for biofilm formation.^58^ Finally, the risk of reinfection increases with each additional revision surgery.

In all treatment scenarios, success is dependent on complete removal of infected and devitalized tissue and hardware alongside an array of possible patient risk factors. Unfortunately, there is no definitive way to ascertain if debridement is successful or not, as dormant and biofilm-dwelling bacteria often cannot be completely removed using this procedure. More importantly, specific guidelines for treating at risk populations are not uniform. As a result, the incidence of infection recurrence following PJI revision is extremely high following all treatments. In a study investigating outcomes of 16 622 TKR infections, 20.8% were treated with incision and drainage (I&D), 15.9% with I&D and liner exchange, 22.7% with single-stage revision, 39.7% with two-stage revision, and 0.98% with amputation.^59^ The results of this study showed that patients who underwent I&D had the highest rate of infection recurrence (28.2%), whereas patients who underwent two-stage revision had the lowest rate of recurrence (19%) at 1 year.^59^ Regardless of the treatment chosen, rates of reinfection are alarmingly high, suggesting the need for continued research on novel diagnostics and therapeutics to combat recurrent infection.

## New definitions of “biofilm” in chronically infected bone

**Fig. 2:**
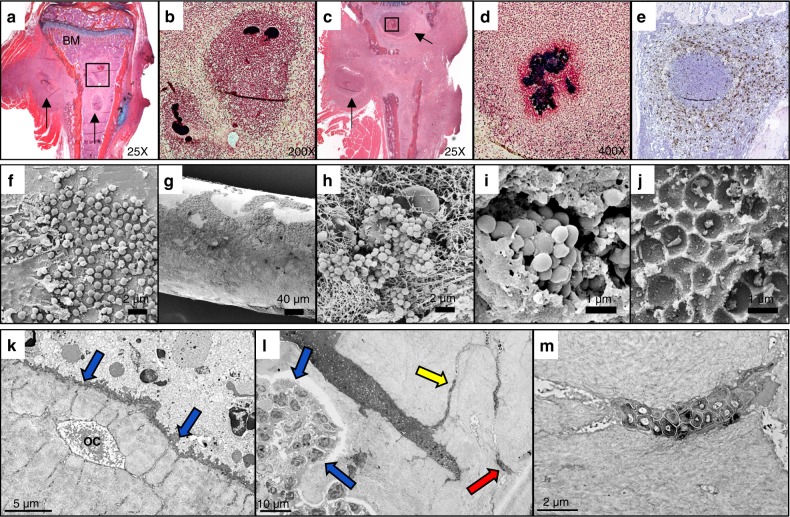
Three distinct reservoirs of bacteria in chronic osteomyelitis. Chronic implant-associated osteomyelitis was established in mice with *S. aureus* as previously described,^84,93,117,267^ and the bacterial burden: (1) in *Staphylococcus* abscess communities (SACs) assessed by histology (**a**–**e**), (2) on the implant assessed by SEM (**f**–**j**), and (3) in cortical bone assessed by TEM (**k**–**m**) is shown. Micrographs of orange G/alcian blue-stained histology of tibiae 7 days (**a**) and 14 days (**c**) post-infection are shown highlighting SACs (arrows) in the bone marrow and adjacent soft tissues. The boxed regions in (**a**), (**c**) are shown in Brown and Brenn-stained parallel section (**b**, **d**) to highlight the Gram+ bacteria (dark blue) surrounded by dead and dying neutrophils following NETosis (red cells), which are surrounded by a ring of macrophages (white layer). Chronic infection is clearly established by day 14, as evidenced by the complete replacement of hematopoietic bone marrow (BM) with inflammatory tissue, and the presence of M2 macrophages (brown cells) surrounding the SAC, as seen by immunostaining with antibody against arginase-1 (**e**). Biofilm formation on the implant commences with planktonic bacterial adhesion (**f**), as illustrated in this case of in vitro *S. aureus* attachment onto a stainless-steel wire incubated in a flow chamber system (×10 000). Following transtibial implantation, the planktonic bacteria rapidly transition to biofilm (**g**), seen as uniform glycocalyx coating the stainless-steel pin 14 days post-op (×200). High power images of the biofilm on the implant reveal cocci adhering to fibrin strands (**h**, ×2 500), and clusters of *S. aureus* forming bacterial pods (**i**, ×5 000). By day 14 post-infection, bacterial emigration from the pod is complete, as evidenced by the empty lacunae (**j**, ×30 000). *S. aureus* colonization of cortical bone commences with eradication of bone lining cells to expose canaliculi (blue arrows) leading to an embedded osteocyte (OC) (**k**, ×6 000). Subsequently, *S. aureus* invasion and propagation through osteocyte lacuno-canalicular network (OLCN) renders the biofilm bacteria (*) inaccessible to activated neutrophils outside the bone (blue arrows) (**l**, ×1 800). The uninhibited bacteria demineralize and consume the cortical bone to expands a canaliculus, and propagate into neighboring canaliculi (yellow arrow), to reach a distant osteocyte (red arrow). **m** High power TEM (×12 000) of the osteocyte in (**l**) killed by *S. aureus* bacterial occupation of its lacunar space

Due to the absence of an effective treatment for progressive osteomyelitis, there is a profound need for research to elucidate the mechanism of recurrent *S. aureus* osteomyelitis. To this end, recent breakthroughs have identified three pathogenically distinct reservoirs of biofilm bacteria in osteomyelitis (Fig. 2), each of which must be treated effectively or the bone infection will likely persist or recur.^60^

### *Staphylococcal* Abscess communities (SACs)

SACs are most commonly observed in skin,^61^ but can be formed in virtually every tissue type following dissemination.^62^ Typically described as a host-induced mechanism for infection control, abscess formation is actually a dynamic process controlled by both host and pathogen signals. While innate immune cells actively sequester infected tissue and associated pathogens within an abscess, in doing so, they confer protection to pathogens at the core of the abscess.^63^ The formation of an abscess creates a physical barrier that prevents immune cell penetration and facilitates long-term bacterial persistence. As a result, abscesses rarely resolve without exogenous antimicrobial treatment.^64^

Peri-prosthetic abscess formation begins with neutrophil recruitment to the site of an acute infection, stimulated by the local release of cytokines and chemokines by host cells. Activated neutrophils proceed to combat extracellular bacteria by phagocytosis, degranulation, and generation of neutrophil extracellular traps (NETosis).^65^ Concurrently, *S. aureus* is capable of attacking neutrophils and other phagocytes by releasing cytolytic toxins, which create pores in host cell membranes.^66^ *S. aureus* also resists host cell attack via an array of immunosuppressive mechanisms,^67,68^ most notably chemotaxis inhibitory protein of staphylococci (CHIPS) and staphylococcal complement inhibitor (SCIN), which modulate polymorphonuclear neutrophil (PMN) killing^69^ and alter macrophage phenotype from bactericidal M1 to anti-inflammatory M2 macrophages.^70^

SACs can begin to form as early as 4 days following infection.^71^ In early stages of SAC formation *S. aureus* coagulases, CoA, and von Willebrand factor-binding protein (vWbp) promote activation of prothrombin and cleavage of fibrinogen,^71^ while membrane-bound protein clumping factor A (ClfA) binds fibrinogen to promote the formation of a fibrin margin at the periphery of the abscess.^72^ The center of a SAC is comprised of live bacteria surrounded by viable and necrotic PMNs separated from healthy tissue by the fibrin margin lined with macrophages to prevent bacterial dissemination,^64^ which is easily detected by 7 days post-infection (Fig. 2a, b). As the SAC matures over weeks, immune cells are restricted to the periphery, where they cannot access bacteria that continue to replicate at the center of the abscess (Fig. 2c, d). M2 phenotype macrophages, which are necessary for resolution of inflammation by efferocytosis of cellular debris and neutrophil extracellular traps (NETs),^73^ are also restricted to the periphery of the abscess, allowing continued bacterial persistence within the abscess (Fig. 2e).

For these reasons, eradication of SACs cannot be accomplished by the immune system alone. Without intervention, SAC persistence within a host can lead to eventual rupture and subsequent bacterial dissemination to new, uncolonized tissue.^63^ Therefore, complete eradication of implant-associated infections relies, in part, on the careful surgical debridement of soft tissue and bone marrow to remove all abscesses harboring bacteria, along with local antimicrobial therapy to ensure elimination of all extracellular bacteria.

### Glycocalyx on the implant

Implant-associated *S. aureus* osteomyelitis begins with bacterial cell adhesion to extracellular matrix components known as Microbial Surface Components Recognizing Adhesive Matrix Molecules (MSCRAMMs).^74^ As the acronym suggests, MSCRAMMs are proteins localized at the microbial cell surface that are capable of binding ligands found in the extracellular matrix, such as fibronectin, fibrinogen, and collagen.^75^ Adhesion of the bacterial cells to a substrate allows for subsequent proliferation and colonization of the region. The initial host innate immune response to bacterial colonization begins with the recognition of pathogen-associated molecular patterns (PAMPs) by pattern recognition receptors (PRRs),^76^ which stimulates the release of an array of pro-inflammatory cytokines, chemokines, antimicrobial peptides, and triggers neutrophil recruitment to the site of infection. At this time, neutrophils and other first responder innate immune cells are capable of targeting and sequestering planktonic bacterial cells by antibody-mediated opsonophagocytosis or killing by oxidative burst through the release of reactive oxygen species (ROS). At some point, PMN attack subsides when all planktonic bacteria are eliminated, or they become inaccessible in biofilms. One of the most notable virulence mechanisms of *S. aureus* is its ability to form biofilm matrices embedded with phenotypically altered bacterial cells.^77^ As an adaptive response to environmental stress, unicellular bacteria often form biofilms to survive in a protected, multi-cellular community that is resistant to host immune response and antibiotic therapy.

Biofilm formation occurs in four generalized stages including: (1) bacterial cell attachment, (2) proliferation, (3) biofilm maturation, and (4) detachment. First, bacteria will attach to a substrate, such as an implant material or segment of necrotic bone, via surface adhesin molecules (Fig. 2f). Then, the attached cells will proliferate to expand the bacterial population and begin producing a matrix of extracellular polymeric substances (EPS) to protect the multi-layered community (Fig. 2e). EPS is generally composed of self-produced polysaccharides, proteins, and nucleic acids (Fig. 2h), which encase the bacterial cells to mediate adhesion, provide mechanical stability and retain essential nutrients and enzymes.^78^ As the biofilm matures, it becomes a complex and heterogeneous structure containing protected pods of bacteria (Fig. 2i), as well as void spaces and intricate channels to facilitate nutrient and oxygen transfer through the bulk of the biofilm. Finally, biofilm detachment or dispersal is a key step in pathogenesis, enabling bacterial cell metastasis via fluid flow to new and uncolonized regions of the host (Fig. 2j).

*S. aureus* biofilms are regulated, in part, by the accessory gene regulator (*agr*) quorum sensing system. The *agr* regulon is a well-described two-component peptide quorum-sensing system that utilizes cell–cell communication to facilitate dynamic expression of an array of virulence genes, including those responsible for biofilm formation.^79,80^ During the initial phase of biofilm formation, the *agr* system responds to the local abundance of self-produced autoinducing peptides (AIPs)^81,82^ by expressing genes associated with colonization including protein A, coagulase, and fibronectin-binding protein^83^ resulting in robust biofilm formation.^79^ As the concentration of AIPs reaches a threshold, the *agr* system activates most temporally expressed virulence genes including, alpha-toxin, beta-hemolysin, Toxic shock syndrome toxin (TSST-1), and leukotoxin, thus activating biofilm dispersal.^84,85^

Biofilms confer long-term bacterial cell survival in hostile environments by a variety of mechanisms. First, biofilms protect bacterial cells from immune onslaught by providing a physical barrier that limits immune cell penetration, thereby preventing phagocytosis and ROS killing. Additionally, bacteria residing within biofilms are particularly pathogenic because of the extreme phenotypic diversity that exists among them, enabling antimicrobial resistance. *S. aureus* in a biofilm can display variable growth rates, altered oxygen, and nutrient dependence and acquired virulence mechanisms via horizontal gene transfer.^86^ For these reasons, bacteria within biofilms become very resistant to antimicrobial treatment and can survive drug dosing up to 1 000 times greater than their planktonic phenotype.^87^

In addition to conferring long-term bacterial survival, biofilms can cause immense damage to the surrounding host tissue. Bacterial infections have been shown to elicit bone resorption, both indirectly through host inflammatory factors and directly through bacterial factors. In the infected bone environment, PAMPs interact with innate toll-like receptors (TLRs) expressed on a variety of cell types and stimulate the release of inflammatory cytokines including: TNF, IL-1, and IL-6.^60^ This leads to activation and differentiation of osteoclasts, while stimulating increased RANKL production by osteoblasts shifting the RANKL/OPG ratio in favor of bone resorption.^88^ Recent studies have shown that host signaling through MyD88 and IL-1R is necessary for the control of infection but paradoxically contributes to pathological bone loss.^89^ Furthermore, ex vivo and in vitro studies have shown that various bacterial organisms have the ability independently degrade bone tissue in the absence of all host factors.^90,91^

Taken together, biofilm presents a significant concern to surgeons when treating implant-associated infections. It is clear that complete eradication of biofilm from implant hardware and associated necrotic tissue is of utmost importance for successful clearance of infection.

Unfortunately, physical removal of biofilm from implant hardware via irrigation or sonication has been met with limited success.^92^ Therefore, complete hardware exchange and extensive debridement remains the gold standard to reduce the risk of reinfection due to bacterial persistence in biofilms.

### Colonization of the osteocyte-lacuno canalicular network (OLCN)

The third and most recently discovered reservoir of bacteria in chronic osteomyelitis is *S. aureus* colonization of the OLCN.^93,94^ This phenomenon was first observed in a systematic examination of infected bone by transmission electron microscopy (TEM) in an experimental murine model of chronic *S. aureus* osteomyelitis.^93^ TEM micrographs showed *S. aureus* invasion of canaliculi perpendicular to the medullary canal and subsequent colonization of lacunar spaces, devoid of osteocytes. These observations do not conform to the historical dogma that defined *S. aureus* as non-motile cocci ~1 μm in diameter, incapable of invading tissue at a submicron scale. Most recently, invasion of the OLCN has been confirmed in clinical bone samples from patients with diabetic foot ulcers and underlying osteomyelitis.^94^

*S. aureus* colonization of the OLCN was shown to be an active process rather than dormant persistence in an experiment that used BrdU labeling of actively dividing *S. aureus* at the leading edge of canalicular invasion.^93^ Based on the absence of any observable motility structures, such as flagella, cilia, or pseudopods, it is theorized that *S. aureus* is capable of invading the canaliculi of live bone with a novel motility mechanism. Our working model of OLCN invasion begins with the eradication of bone lining cells from cortical bone due to local inflammation and necrosis during the establishment of *S. aureus* infection (Fig. 2k). Next, we theorize that *S. aureus* can identify exposed submicron canalicular orifices via haptotaxis, triggering asymmetric binary fission in which deformed daughter cells are extruded. Widening and scalloping of colonized canaliculi is frequently observed, as the bacterial acid demineralizes the bone, and degradative enzymes consume the organic bone matrix for nutrients (Fig. 2l), a phenomenon also observed in ex vivo studies independent of host factors.^90^ Invasion proceeds with proliferation of *S. aureus* cells through canaliculi and lacunar spaces as osteocytes are killed by bacterial colonization (Fig. 2m). Unlike bacteria in the bone marrow that are surrounded by neutrophils, *S. aureus* inside the OLCN are surrounded by the dense mineral matrix of cortical bone and are completely inaccessible to immune cells (Fig. 2l). These observations suggest that *S. aureus* may be able to survive for decades within the OLCN with an inexhaustible supply of nutrients, while evading immune attack.

Recently, an in vitro platform was developed to mimic *S. aureus* invasion of submicron spaces, like that of the OLCN, using a nanoporous membrane.^95^ Surprisingly, this study found that *S. aureus* propagation through nanopores in vitro is not dependent on activation of the *agr* quorum sensing system. This result was validated in vivo where *agr* deletion did not impair the bacteria’s ability to invade the submicron-sized canalicular network of cortical bone despite global down-regulation of virulence-associated genes. Taken together, it is clear that continued research is warranted to determine the genetic mechanisms of *S. aureus* invasion of the OLCN.

The discovery of OLCN invasion is particularly concerning for the treatment of implant-associated osteomyelitis, because it is impossible to know if all segments of infected bone have been completely debrided. Debridement of bone is guided by the presence of necrotic “white” bone and healthy “red” bone. Directed by this method alone, *S. aureus* colonized bone could remain in the patient despite extensive debridement and eventually facilitate recurrence of infection following revision surgery. Additionally, deep invasion of cortical bone by bacteria occurs within 2 weeks in our mouse models of osteomyelitis, which suggests this hallmark of chronic infection may actually occur within the timescale of acute osteomyelitis.

Eradication of bacteria infecting the OLCN of bone remains an open question as it is currently not known if standard-of-care antibiotic therapy can be effective against *S. aureus* embedded within the bone matrix. Furthermore, it is possible that the bacteria within the OLCN exhibit an altered growth phenotype like that of SCVs making them tolerant to antimicrobial therapy. Recent studies have indicated that only select antimicrobials are effective against SCVs,^96^ thus additional experiments to confirm the efficacy of antibiotic therapy on *S. aureus* within the OLCN are needed.

Collectively, these novel findings suggest that invasion and propagation within the immune-privileged OLCN is a pathogenic mechanism that renders *S. aureus* infection of bone incurable. Further, the long-term colonization of cortical bone by slow growing bacteria seems to be the most likely explanation for infection recurrence after decades of quiescence.^32,33,97^ Considering this new information on distinct biofilms in chronic osteomyelitis, including SACs and colonization of the OLCN of live bone, a major unmet clinical need is a diagnostic that can identify the existence of bone infection with spatial resolution, and assess the completeness of debridement following revision surgery. Indeed the 2018 ICM Biofilm Workgroup deliberated on the question of, “Is the mapping of biofilm to a particular component or anatomical location an important consideration in management of implant-related infections?”^8^ Unfortunately, this group of experts concluded that, “At present, mapping of biofilms is only possible in the laboratory, not in the clinical setting. Therefore, it is of unknown clinical importance in relation to management of implant‐related infections.” Interested readers are encouraged to review these proceedings online.^13^

## The role of *S. aureus* intracellular persistence in osteomyelitis

As mentioned above, the interspersed periods of bacterial quiescence in chronic osteomyelitis has been a perturbing problem that is readily documented over the years.^32,33,97^ A particularly interesting case of *S. aureus* osteomyelitis described a woman who was initially infected in her left femur as a child and treated surgically without antibiotic therapy.^97^ Approximately 75 years later the infection returned, and genetic analysis of the infecting pathogen revealed the *S. aureus* to be of the same strain from 75 years prior, maintaining its sensitivity to penicillin and oxacillin. This case demonstrates an interesting immune evasion strategy in which a single clonal strain of *S. aureus* can remain quiescent for long periods of time, then awaken decades later to cause serious osteomyelitis.

In addition to the mechanisms of osteomyelitis persistence described above, many groups have questioned the contribution of intracellular colonization of bone cells as a possible mechanism for quiescent osteomyelitis. The role of intracellular persistence in chronic osteomyelitis disease pathology remains a subject of intense debate because of a lack of compelling in vivo evidence.^98,99^

Intracellular persistence of *S. aureus* in a variety of host cell-types has been described in many disease settings.^98–100^ Current literature has shown that *S. aureus* is capable of surviving within professional and non-professional phagocytes including, macrophages, neutrophils, fibroblasts, keratinocytes, epithelial cells, and endothelial cells.^101,102^ Internalization can be triggered through initial attachment to host cell surface-associated proteins using MSCRAMMs. For example, fibronectin-binding proteins A and B (FnBP) have been shown to initialize internalization of *S. aureus* via fibronectin bridging to α_5_β_1_ integrins and subsequent cytoskeletal reorganization in endothelial cells.^103^ Once internalized, the bacteria can survive cell death by preventing fusion of the phagolysosome, escaping the endosome into the cytoplasm or resistance to cellular enzymes.^104,105^

In the context of osteomyelitis, osteoblasts have been the primary host cell investigated for intracellular persistence because of their long life-span and ultimate differentiation into osteocytes. Some groups hypothesize that osteoblasts^106,107^ can be infected intracellularly, resulting in persistence in chronic osteomyelitis, based on in vitro findings. These studies investigated many factors such as osteoblast cell signaling,^108,109^ induction of osteoblast apoptosis,^110^ the effect of antibiotics on intracellular *S. aureus*,^107,111,112^ and bacterial cell gene expression.^113^ Hamza et al. found that osteomyelitis could be induced in vivo by infecting the femurs of rats in an open femoral fracture model using osteoblasts that were previously intracellularly infected in vitro.^114^ The authors claim that this study provides direct in vivo evidence of osteomyelitis induction by intracellular infection alone. However, no difference between induction of osteomyelitis with intracellular and extracellular *S. aureus* were observed as establishment of infection required the exact same inoculum of 10^2^ CFU *S. aureus* in both groups. Further, it cannot be determined if an infection was truly established by intracellularly infected osteoblasts, or if osteoblasts containing *S. aureus* simply lysed upon implantation in the host, releasing bacteria extracellularly for the establishment of infection.

Other bone cells such as cultured osteoclasts^54^ and osteocytes^115^ have been studied in vitro and demonstrate the ability to internalize *S. aureus*. While these studies may describe essential details on the interactions between bone cells and *S. aureus*, they do not necessarily provide direct evidence of intracellular infection in vivo.

The current research on intracellular persistence of *S. aureus* in osteomyelitis in vivo is still very limited. A small number of clinical case studies have shown intracellular persistence, where one study identified fibroblasts with internalized *S. aureus* in a PJI case,^29^ and another described a patient with recurrent osteomyelitis with intracellularly infected osteoblasts and osteocytes shown by TEM.^33^ The only experimental study that demonstrated intracellular infection of bone cells in vivo was performed 2 decades ago, in which embryonic chicks were infected with *S. aureus* and bone samples interrogated by TEM showed bone cells with internalized *S. aureus*.^116^

**Fig. 3:**
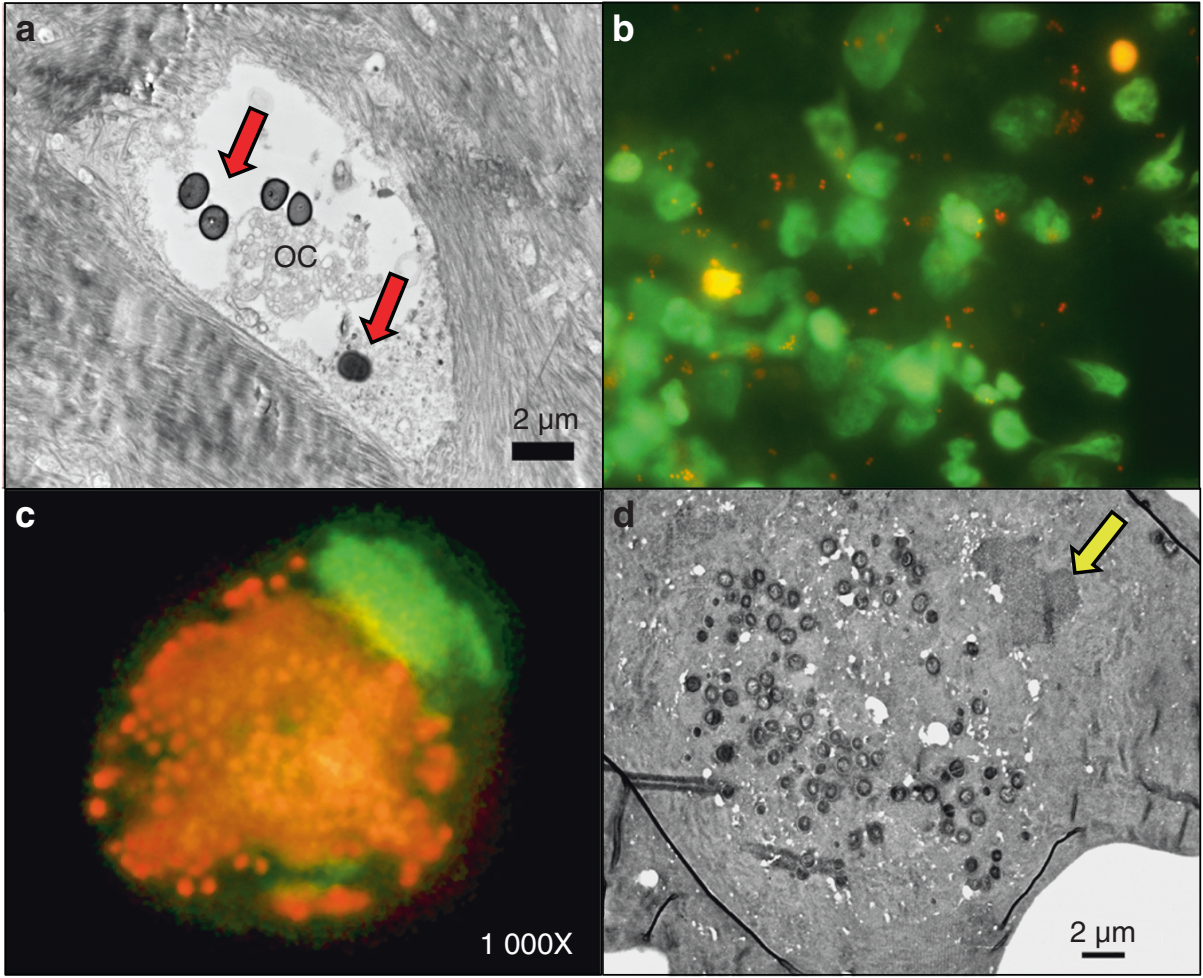
The role of *S. aureus* intracellular infection as a virulence mechanism in chronic osteomyelitis. Extensive TEM analyses of *S. aureus-*infected human bone samples (*n* > *X*) failed to identify significant evidence of viable bone cells (osteoblasts, osteoclasts, osteocytes) containing intracellular bacteria, while all *S. aureus* colonized OLCN contain necrotic osteocytes (OC) with extracellular bacteria (red arrows) within osteocyte lacunae (**a**, TEM ×15 000). In contrast, an acridine orange-stained smears of blood, harvested post-mortem from a patient that died from septic multiorgan failure, demonstrates both extracellular bacteria (orange) and colonized leukocytes (yellow cells) via fluorescent microscopy (**b**, ×20). **c** A higher power fluorescent image of the blood smear reveals a “Trojan horse” macrophage with cytoplasmic *S. aureus*, and acentric nucleus (fluorescent green). **d** TEM (×20 000) of this Trojan horse macrophage was performed via a “pop-off” technique, which confirmed intracellular *S. aureus* cocci within the cytoplasm, adjacent to the nucleus (yellow arrow)

We have studied the pathogenesis of *S. aureus* chronic osteomyelitis extensively in experimental in vivo models, as well as in clinical cases.^84,93,94,117^ Throughout all in vivo studies, we failed to identify intracellularly infected live bone cells. Using TEM examination as described above, *S. aureus* invasion of the OLCN of cortical bone is consistently observed with exclusively dead or dying infected bone cells (Fig. 3a). As a result of these expansive studies, we conclude that intracellular persistence within osteoblasts, osteoclasts, and osteocytes is not a meaningful mechanism of chronic or recurrent osteomyelitis. In contrast, we routinely observed intracellular infection of leukocytes, so called “Trojan Horses,”^100^ in septic animals and patients. This occurs via *S. aureus* invasion without triggering a bactericidal respiratory burst, creating circulating cells that facilitate bacterial metastasis to immune privileged sites.^118^ Intracellular persistence within macrophages is particularly concerning because of this leukocyte’s longer life span and ability to travel through the host circulatory system.^119^

In addition to persistent and recurrent infection, sepsis is a significant concern in implant-associated osteomyelitis. Approximately, 10% of PJI cases lead to sepsis and death,^12^ and *S. aureus* remains the leading and most deadly cause of bacteremia in the United States.^120^ In a postmortem examination study of patients who died from *S. aureus* septic multi-organ failure, fluorescent imaging of acridine orange-stained blood smears showed both extracellular bacteria as well as intracellularly colonized leukocytes (Fig. 3b). Higher magnification fluorescent and electron microscopy imaging revealed *S. aureus* survival within the cytoplasm of a Trojan Horse macrophage (Fig. 3c, d).

In a study directly comparing osteoblast and macrophage infection with *S. aureus*, researchers found that macrophages contained 100-fold more live bacteria than osteoblasts, and that osteoblasts were significantly less viable.^121^ This result suggests that macrophages may be a more likely host cell candidate for *S. aureus* intracellular persistence. Further, the same study found that *S. aureus* only survived within osteoblasts and macrophages for 7 and 5 days,^121^ respectively, suggesting that these cell types are likely not the source of persistent osteomyelitis years after initial infection.

It remains possible that intracellular infection could play a role in bone infection, however, that role is not immediately apparent because of the extremely limited amount of in vivo evidence. One could hypothesize that *S. aureus* may survive intracellularly for a brief period of time to avoid innate immune attack at early stages of infection, potentially providing direct access to exposed canaliculi on the bone surface. To that end, we are currently exploring the virulence mechanisms that may promote intracellular survival of *S. aureus* in the OLCN.

Collectively, it is apparent that additional in vivo studies must be performed to elucidate the specific role of *S. aureus* intracellular persistence in chronic osteomyelitis. A successful in vivo experiment investigating intracellular infection should be able to demonstrate that the host cells along with internalized bacterial cells, are both alive for a prolonged period of time. Further, a successful experiment should demonstrate that internalized bacterial cells have the same clonality as the initial infecting strain. Currently, most analyses of intracellular persistence are performed using TEM, and are therefore limited in their ability to define whether cells are alive or dead.^122^ Advances in in vivo imaging techniques^123^ combined with clinically relevant animal models^124^ may allow for more accurate longitudinal tracking of bacterial infections to possibly answer some outstanding questions related to intracellular persistence in osteomyelitis.

## Classification of acute and chronic osteomyelitis

Many factors can increase the risk of osteomyelitis including: diabetes, obesity, malignancy, immune deficiencies, substance abuse, malnutrition, and trauma.^41,125^ Due to large variations in patient populations, bimodal age distribution (most patients are younger than 20 or older than 50) and variable clinical presentation, classification guidelines for selecting appropriate treatment are widely variable and not ubiquitously accepted.

Detection of osteomyelitis in the clinic relies on a combination of techniques including: clinical signs, radiology,^126^ and wound swabbing with organism culture or polymerase chain reaction (PCR) for species identification. Once detected, the diagnosis of osteomyelitis is commonly categorized into subjectively defined groups of sub-acute, acute, or chronic stages of disease severity.

In its simplest form, osteomyelitis classification will rely on disease duration to suggest a specific course of treatment. Acute osteomyelitis typically describes a recent bone infection that causes systemic inflammation.^55^ Chronic osteomyelitis describes bone infection of longer duration, with minimal systemic symptoms and the presence of key pathological features, such as a lymphoplasmacytic infiltrate, marrow fibrosis, and reactive new bone formation. Chronic stage osteomyelitis is sometimes defined as early as 4 weeks,^50^ since initial disease presentation to as late as 6 months^127^ and generally requires surgery.^55^ However, there is a lack of scientific rationale for the selection of a specific time-point causing a lack of consensus among medical professionals.

Other methods of classification, accessory to disease duration and clinical signs, include modalities of radiographic imaging.^128,129^ Radiography plays a vital role in the diagnosis of osteomyelitis and can be used to evaluate the extent of infection, the anatomy involved and even specific hallmarks of acute vs. chronic stage disease, such as the presence of a sinus tract, sequestrum (avascular necrotic bone), or involucrum (new bone formation).^130^ Unfortunately, typical radiographic techniques are limited in their accuracy when detecting low level disease, as well as in the presence of metallic implants, which decrease image quality with artifact.

When available, an intraoperative histopathological examination can be used to identify regions of acute infection in periprosthetic tissue specimens. Past studies have shown that acute inflammation defined by the presence of >1–10 polymorphonuclear leukocytes in several high-power fields of view (400×) can be a useful predictor for acute infection.^131–133^ However, the variability of osteomyelitis disease presentation extends beyond patient-to-patient differences to variability on a cellular level within a single lesion.

**Fig. 4:**
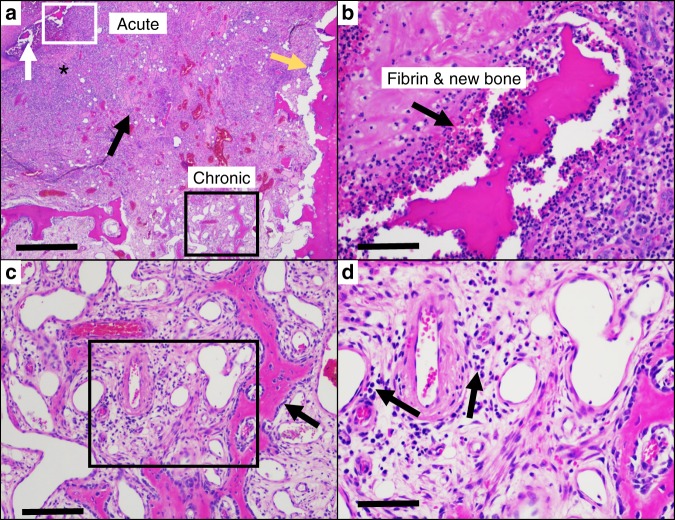
Histologic features of “acute” and “chronic” osteomyelitis exist in the same lesion. Hematoxylin and eosin-stained, paraffin-embedded, decalcified section of an infected metatarsal bone resected from a patient with a diabetic foot ulcer is shown, illustrating salient features of both acute and chronic osteomyelitis in the same bone. **a** Low power micrograph of the lesion in which most of the trabecular bone in this part of the metatarsus has been destroyed and replaced by an acute inflammatory reaction, consisting of neutrophils (*) and fibrovascular granulation tissue (black arrow) (scale bar = 1 mm). The inflammation extends to the bone beneath the articular cartilage (yellow arrow) and has destroyed much of the cortical bone (white arrow). Reactive new bone has formed in the lower part of the image, along with a chronic inflammatory and fibrovascular reaction. **b** A region of interest of acute inflammation (white box in **a**) is shown highlighting a fragment of dead cortical bone surround by neutrophils (black arrow), with an associated fibrinous exudate, which are hallmarks of acute osteomyelitis (scale bar = 25 mm). **c** A region of interest of chronic inflammation (black box in **a**) showing new bone formation (black arrow), and replacement of normal bone marrow with fibrovascular inflammatory tissue (boxed region) (scale bar = 50 mm). **d** This region of interest (boxed area in **c**) is presented at high power, showing blood vessels, osteoblasts rimming newly formed woven bone (bottom right), and collections of lymphocytes and plasma cells (arrows), which are characteristic of chronic osteomyelitis (scale bar = 25 mm)

When a segment of infected bone is investigated histologically for hallmarks of acute and chronic inflammations (Fig. 4), it becomes clear that features of both acute and chronic osteomyelitis can be present in the same specimen. From a case of acute osteomyelitis in a tarsal bone under an infected ulcer in a diabetic patient, we can observe distinct characteristics of acute infection, as well as chronic inflammation within the same lesion (Fig. 4a). A region of acute infection can be identified by the presence of predominantly newly activated neutrophils, as well as fibrovascular granulation tissue around foci of dead bone (Fig. 4b). However, adjacent to this lesion, we can identify hallmarks of chronic inflammation where normal bone marrow is replaced by fibrosis, reactive bone formation, blood vessels and collections of lymphocytes and plasma cells (Fig. 4c, d). This case highlights the significant amount of heterogeneity that can exist within a single osteomyelitis lesion and is typical of many infected foot ulcers with underlying osteomyelitis that has been treated conservatively before surgery.

Collectively, empiric definitions of acute and chronic stage osteomyelitis fail to accurately and comprehensively describe the extent of infection. Such classifications of acute vs. chronic stage disease significantly diminish guidance for treatment. For these reasons, we recommend that time-dependent classifications of osteomyelitis should be avoided because of extreme patient-to-patient variability, as well as pathophysiological variability at the cellular level.

Lack of definitive methods to effectively discriminate acute vs. chronic-stage bone infections without biopsy and histologic examination makes treatment protocols based on such classifications almost meaningless. It should be assumed at all stages of prosthesis infection that the patient is at risk for soft tissue abscess formation, biofilm formation on implant hardware, and cortical bone colonization by bacteria.

Alternative classification systems based on criteria, such as the condition of the host, extent of disease^134^ and previous disease admission^135^ have been proposed to guide and improve the success of disease treatment. While these classifications have achieved minimal traction and fail to achieve consensus among medical professionals, they demonstrate constructive progress in the definition of osteomyelitis. Therefore, although the time has come to update the clinical definitions of “acute versus chronic” bone infection, as this issue was identified as “the greatest research priority from 2018 International Consensus Meeting” (2018 ICM) on Musculoskeletal Infection,^13^ these key opinion leaders also found this issue to be the most controversial topic in this field. Remarkably, three attempts to draft a recommendation for the question, “What is the recommended time interval that would divide acute and chronic peri-PJI (4 weeks, 90 days, etc.)?” failed, and interested readers are encouraged to review these proceedings online.^13^ Thus, given the complexities that surround this issue, we cannot offer new definitions at this time. However, we concur with the 2018 ICM Workgroup that “these new definitions must incorporate our current understanding that the management of bone and implant‐related infections is inextricably intertwined with the biology of biofilms, infection of osteoblasts and osteocytes, and invasion of the osteocytic‐canalicular network by the bacteria.”^13^

## Antibodies and *S. aureus* orthopedic infections

*S. aureus* has evolved numerous strategies to efficiently evade adaptive host defenses, which consist of cell-mediated responses dominated by T-cells and humoral antibody responses mediated by B-cells. Anti-*S. aureus* antibodies are prevalent in all humans due to life-long exposure to *S. aureus* by means of prior infections or asymptomatic carriage.^136–140^ However, the presence of these antibodies in the host (collectively termed humoral “immune proteome”) does not necessarily warrant protection against future infections. In fact, some individuals are more susceptible to *S. aureus* PJI than others likely due to the protective vs. susceptible nature of their immune proteome. Thus, a better understanding of the functional role of specific antibodies in *S. aureus* infections can aid in predicting outcomes, while providing targets for the development of novel therapies to combat *S. aureus* bone infection. In this section, we will discuss the utility of antibodies in diagnosing *S. aureus* orthopedic infections in addition to summarizing the discovery and development of antibody-based biologics to manage *S. aureus* infections.

### Antibodies as diagnostic and prognostic markers of orthopedic infections

Deep-rooted infections such as implant-associated osteomyelitis present a diagnostic predicament because patients typically present with non-specific symptoms, including pain, swelling of the joint, and fever. Definitive diagnosis of the infection requires obtaining intraoperative specimens or via image-guided biopsy procedures, which are expensive, invasive, and traumatic. Most clinicians rely on inflammatory cell counts and on markers, such as the C-reactive protein (CRP) and erythrocyte sedimentation rate (ESR) for diagnosing *S. aureus* orthopedic infections. However, these techniques are not pathogen-specific, nor are they anatomically specific. Importantly, they cannot accurately assess if a patient is responding to treatment.

Blood-based approaches describing anti-*S. aureus* humoral immune response in physiologic and pathologic conditions provide superior alternatives for diagnosing *S. aureus* infections. Indeed, several groups including ours have attempted to describe blood-serum-based *S. aureus*-specific antibodies during colonization, bacteremia, and orthopedic infections.^138,139,141–144^ Utilizing a multi-antigen Luminex immunoassay with immunodominant antigens belonging to distinct functional classes, such as iron acquisition proteins, cell division proteins, secreted toxins, and cell attachment proteins, we demonstrated that: (1) *S. aureus* orthopedic infections can be diagnosed using antibody-based immunoassays, (2) anti-*S. aureus* antibody responses against certain antigens predominate during PJI, and (3) single antigen and/or a combination of antigens are useful predictors of ongoing *S. aureus* orthopedic infection.^144^ For instance, our studies illustrated that antibody responses against iron-sensing determinant proteins (IsdA, IsdB, IsdH) could be predictors of infection outcomes in patients with *S. aureus* PJI, and that higher serum anti-IsdA and anti-IsdB IgG levels in patients were associated with increased mortality.^144^ In contrast, IgG levels in serum against a cell division protein called autolysin (Atl) was protective against *S. aureus* infections in patients undergoing orthopedic surgeries.^117,143^

The clinical utility of serum-based immunoassays for the diagnosis of microbial infections cannot be discounted. However, high levels of pre-existing anti-*S. aureus* antibodies are present in most patients due to asymptomatic carriage.^136,139^ Additionally, prior exposure due to infection confounds reliable diagnosis of *S. aureus* infections using this method. One way to obviate this problem is by examining pathogen-specific antibodies in recently stimulated circulating plasmablasts in the patient’s blood.

As adaptive responses develop during an infection, pathogen-specific B-cells stimulated in germinal centers of lymph nodes proliferate, secrete their antibodies and enter the circulation. These plasmablasts, often called antibody-secreting cells (ASCs), emerge early in an infection and are circulating in the blood as long as the infecting pathogen remains active.^145–147^ This attribute makes ASCs and their specific antibodies perfect biomarkers for ongoing infections, successful (or failed) therapy, and recurrence. Though ASCs have been explored in multiple settings,^145–147^ they have yet to be exploited in general medical practice for infection diagnosis and prognosis. Here, we describe a simple approach developed by our group to utilize ASCs for diagnosing *S. aureus* orthopedic infections.

**Fig. 5:**
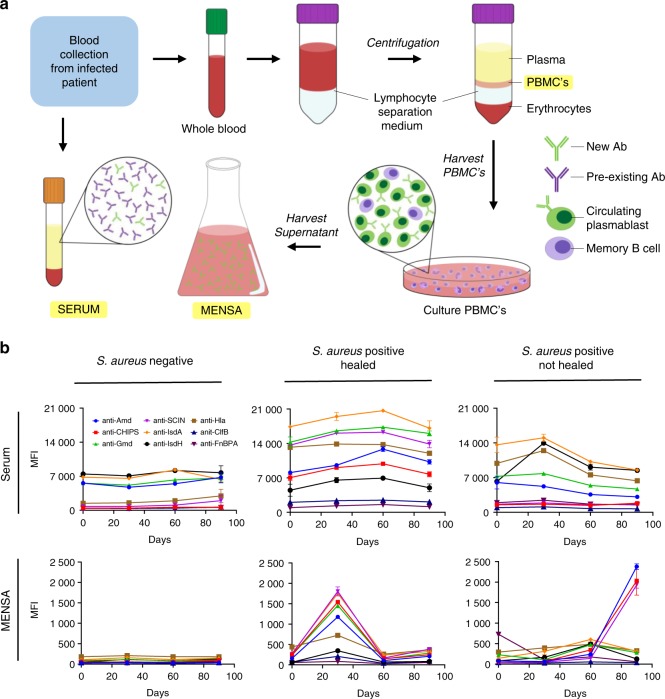
A diagnostic and prognostic immunoassay for the measurement of anti-*S. aureus* antibody levels in patients with osteomyelitis. **a** Schematic illustration of production of serum and isolation of medium enriched for newly synthesized anti-*S. aureus* antibodies (MENSA) from peripheral mononuclear cells of patients with osteomyelitis. **b** Anti-*S. aureus* antibody levels in serum and MENSA were determined using a custom bead-based multiantigen Luminex immunoassay developed by our group. Here, we examined anti-*S. aureus* IgG responses in serum and MENSA of diabetic foot infections (DFI) patients undergoing foot salvage antimicrobial therapy (FST). The change in antibody titers over the course of FST of a representative patient whose DFI was negative for *S. aureus*, a patient with *S. aureus* infection that responded to FST, and a patient with *S. aureus* DFI who failed FST is presented here. Remarkably, MENSA levels faithfully reflected the *S. aureus* infection over time while serum levels remained unchanged (see Oh et al.^148^ for more details)

Peripheral blood mononuclear cells (PBMC), which include newly activated ASCs, are harvested from whole blood and washed extensively to remove pre-existing serum immunoglobulins. Subsequently, the ASCs are cultured in vitro to allow their expression and secretion of antibodies in response to any ongoing infection (Fig. 5a). The resulting media enriched for newly synthesized antibodies (MENSA) represent a measurable and accessible way to detect acute immune responses to ongoing infections. MENSA levels are direct indicators of ongoing infections as they develop and as the infections wane under the attack of the host’s immune responses.

In a recent study involving the management of foot salvage therapy (FST) for diabetic foot infections (DFI), we demonstrated that our immunoassay utilizing MENSA can accurately monitor treatment response and detect persistent or recurrent infections that are invisible in a serum response.^148^ Anti-*S. aureus* IgG levels in MENSA decreased with successful FST, and rose with re-infection, while IgG levels in serum remained unchanged throughout the FST treatment period (Fig. 5b). These results demonstrate that MENSA evaluation could be used as a prognostic tool to guide clinical decisions in orthopedic infections. Additionally, tracking changes within the DFI microbiome may be a promising tool for monitoring treatment response.^149^

### Antibodies as biologics for combatting orthopedic infections

Battling the tenacious *S. aureus* with antibiotics is a challenge because the pathogen has extraordinary abilities to develop resistance against antibiotics.^150^ Immunotherapies may be effective adjuvants with antibiotics for combating hard-to-treat osteomyelitis. Over the past two decades, numerous potential *S. aureus* vaccine targets received preclinical validation,^151,152^ yet none turned out to be effective in providing protection against *S. aureus* infections in humans.^153^ For instance, a phase 2 clinical trial of an IsdB active vaccine (Merck’s V710), in which 8000 patients undergoing heart valve replacement were randomized to placebo or the vaccine, had to be stopped due to a five-fold increase in fatal and adverse outcomes in the vaccinated individuals due to *S. aureus* bacteremia.^154^ This clinical trial and several others were either terminated abruptly by the FDA or showed no efficacy in humans. Major factors contributing to the failure of functional active vaccines against *S. aureus* include: (1) use of antigen targets that produce antibodies, which opsonize bacteria but fail to induce cell lysis or phagocytosis, (2) limited biological activity against the targets, (3) overreliance on murine models for preclinical validation, and (4) lack of strategies to combat site-specific infection such as osteomyelitis.^104,151–153,155–157^

Passive immunization involving monoclonal antibodies (mAbs) is becoming a more attractive immunotherapy to treat *S. aureus* osteomyelitis. A benefit of passive immunization with mAbs is their high antigen-neutralizing specificity. Additionally, mAbs can be administered locally to the site of infection, and production of mAb at scale is not cost prohibitive. Several anti-*S. aureus* mAb passive immunization agents have been evaluated in human clinical trials. These include mAbs that target fibrinogen-binding cell surface protein (ClfA),^158^ cell wall components (lipoteichoic acid (LTA), poly-*N*-acetylated glucosamine (PNAG))^159,160^ and secreted toxin, such as α-hemolysin (MEDI4893 and AR-301).^161–164^ Unfortunately, these first-generation mAb biologics, despite their promise of safety and tolerability in humans, demonstrated limited efficacy in clinical trials. Their inadequate success could again be attributed to limited biological activity against the target, their inability to effectively mediate phagocytic killing, and the array of different mechanisms utilized by *S. aureus* to circumvent components of host immune system.^165,166^

The second generation of antibody-based biologics, currently in preclinical development, target multiple antigens with essential functions for *S. aureus* immunoevasion, colonization, intracellular growth and persistence within the host cells. Targeting leukocidin toxins that selectively kill immune cells is key to attenuating *S. aureus* virulence. Therefore, several multivalent mAbs that can neutralize these toxins are currently under development. For instance, Rouha and colleagues described one such multivalent mAb that targets α-hemolysin and four leukocidins (HlgAB, HlgCB, LukED, PVL). This broadly toxin-neutralizing mAb was shown to provide protection against *S. aureus* in murine sepsis and pneumonia models.^167^ Other neutralizing antibodies that target essential *S. aureus* immunoevasion mechanisms are also under preclinical testing. These include mAbs that target the B-cell antigen staphylococcal protein A,^168,169^ the immunodominant surface protein IsaA,^170^ and the immune-stimulatory staphylococcal superantigen SEB.^171,172^ Interestingly, multivalent mAbs that simultaneously target and neutralize several of the aforementioned toxins and immunoevasion proteins are under preclinical development by various pharmaceutical companies and research institutions.

It has recently been acknowledged that blocking the ability of *S. aureus* to colonize, persist, and grow intracellularly within different host cells, such as macrophages, may be a viable strategy to reduce *S. aureus* burden in human disease. We discovered that certain PJI patients who have high serum IgG levels against a cell division protein called Atl ended up recovering fully from MRSA PJI, whereas those that had lower anti-Atl IgG levels often had morbid clinical outcomes.^143^ To further investigate this intriguing phenomenon, we developed mAbs against the glucosaminidase (Gmd) subunit of Atl. Not surprisingly, we observed that anti-Gmd mAb can inhibit *S. aureus* cellular functions, such as binary fission, induce megacluster formation, and opsonophagocytosis by macrophages, and protect mice from *S. aureus* implant-associated osteomyelitis.^117,173^

Based on the recent transformative clinical success of biologic checkpoint inhibitor cancer immunotherapy, it is likely that antibody-based biologics offer similar promise for treating *S. aureus* osteomyelitis. Encouragingly, there are several anti-*S. aureus* mAb biologics currently under preclinical and clinical development. Combined with our advances in understanding the pathophysiology of *S. aureus*, we optimistically predict that, in time, mAb-based biologics will be routinely utilized to prevent and treat *S. aureus* osteomyelitis.

## Novel antibiotic therapies to combat osteomyelitis

The recurring and resilient nature of osteomyelitis, as well as its affiliated high patient morbidity, extended hospitalization, and costly healthcare expenses, have prompted research efforts to develop novel therapeutics to improve upon the standards used today to treat osteomyelitis. These efforts have primarily focused on developing and investigating implant coatings that inhibit bacterial adhesion, prevent biofilm formation, and provide bactericidal activity, as well as therapeutics for the local delivery of antimicrobials in dead space management. These strategies to combat osteomyelitis are essential to consistently achieve favorable patient outcomes.

### Implant coatings

As discussed previously, *S. aureus* has the ability to attach to and colonize both the tissues of the musculoskeletal system and orthopedic implants.^84,85,174,175^ *S. aureus* first adheres to a substrate by binding to host proteins, including fibrinogen via Clumping factor A and B (ClfA, ClfB), fibronectin via fibronectin-binding proteins A and B (FnBPA, FnBPB), and collagen via collagen adhesin (Can).^75^ Then subsequent colonization, and expression of various virulence factors (i.e. exotoxins, enterotoxins, adhesive factors, etc.)^176,177^ prompt a detrimental inflammatory reaction.^130,178^ In order to halt the ultimate development of PJI, implants are treated with physical and chemical modifications to prevent the first and cardinal step of infection, bacterial adherence. Yet, it is important to note that implant coatings primarily reduce a patient’s susceptibility to infection and are not effective in treating an established infection.

Silver is often studied as an antimicrobial coating due to its broad spectrum activity against Gram-positive and Gram-negative bacteria, fungi, and viruses.^179^ As a result of its nonspecific biocidal activity, silver has been used in various industrial, healthcare, and domestic applications most commonly in the topical chemoprophylaxis of burns either as a topical cream or antimicrobial wound dressing.^180^ The mechanism of action of silver is a result of biologically active ions released from its surface producing a toxic effect.^181,182^ Silver toxicity arises from three mechanisms. First, silver ions bound to the cell wall create morphological irregularities causing cell lysis and release of intracellular contents.^183^ Second, silver ions bound to sulfhydryl groups of proteins or DNA impair essential metabolic pathways and inhibit DNA replication.^184^ And lastly, silver has been observed to produce excessive ROS causing oxidative damage.^185^ These mechanisms of action also prevent biofilm formation by inhibiting the production of exopolysaccharides, which is a required prerequisite for biofilm formation.^186^

Due to its successful application in various fields, silver has been used to directly coat orthopedic implants, added to bone cement^187,188^ and incorporated into hydroxyapatite (HA) coatings of replacements.^189,190^ However, the true efficacy and safety of silver is still debated in orthopedic applications. Incorporation of silver nanoparticles into bone cement has shown increased antibacterial activity against various strains, including *Staphylococcus epidermidis*, methicillin-resistant *S. epidermis* (MRSE), and MRSA, compared to gentamicin-impregnated bone cement.^187^ While bone cement eluting silver nanoparticles does not appear to be cytotoxic to fibroblast cells, silver salt-eluting bone cement is confirmed to be highly cytotoxic.^191,192^ A study investigating the direct application of silver to Kirschner-wires (K-wires) inserted into *S. aureus-*infected femoral canals of rabbits found no difference in bacterial colonization between the coated and non-coated K-wires.^189^ However, the incorporation of silver into HA coatings of metal implants has shown more favorable antibacterial outcomes, with the added benefit of HA composition for improved osseointegration.^193–196^ Akiyama et al. investigated the antimicrobial activity of titanium rods coated with HA or silver (Ag)-HA in a murine model of MRSA osteomyelitis and found that Ag-HA-coated rods showed increased antimicrobial activity and increased bone formation compared to HA-coated rods.^193^

Clinical studies have also shown that coating implants with silver substantially reduces implant-associated osteomyelitis. Wafa et al. reported in a case-controlled study that silver-coated implants had a 11.8% infection rate compared to 22.4% for the uncoated-control group.^197^ Similarly, in another case-controlled study, Hardes et al. reported a 5.9%–17.6% infection reduction in patients with bone sarcoma treated with silver-coated implants.^198^ While these results appear promising, a serious concern for the application of silver in osteomyelitis treatment is systemic^192,199^ and cellular^200,201^ toxicity, documented in clinical cases of neuropathy^192^ and myoclonic status epilepticus.^199^ Because of silver toxicity observed in vitro and in vivo, additional studies are needed to investigate ideal formulations and effective concentrations for the safe clinical use of silver.^202^

In addition to silver, antibiotics have also been thoroughly investigated for incorporation into implant coatings. Antibiotics are the clinical standard for both systemic and local treatment of various infections caused by a broad-spectrum of pathogens and unlike silver, are not considered highly cytotoxic. Therefore, prophylactic coating of implant hardware with antibiotics is a rational approach for infection prevention. Coating hardware with antibiotics enables the local delivery of high concentrations of drug that would otherwise be toxic if delivered systemically. Numerous coating techniques and antibiotics have been investigated to treat *S. aureus*-related bone infections. The most common strategy for antibiotic coating is the attachment of biocompatible synthetic polymers loaded with antibiotics to the surface of implants. Commonly used polymers are poly(d,l-lactide) (PDLLA),^203–207^ polylactic acid (PLA),^208^ polylactic-co-glycolic acid (PLGA),^209,210^ poly(β-amino esters)/poly(acrylic acid),^211^ and poly(ethylene glycol)-poly(lactic-*co*-caprolactone) (PEG-PLC).^212^ Alternative drug-coating techniques include coprecipitation and covalent bonding of drug to implant.^213–215^ Gentamicin is the most studied antibiotic in coatings because of its application in the clinic for treating methicillin-sensitive *S. aureus* (MSSA) bone-related infections.^203,204,206–208,210,211,213,214,216–218^ Vancomycin, commonly used to treat MRSA infections, has also been incorporated into implant coatings.^212,215,219^ Additional antibiotics such as fosfomycin,^204^ doxycycline,^209^ minocycline,^20^ rifampin,^20^ colistin, daptomycin, and cefoxitin have also been incorporated into implant coatings.^205^ Studies using antibiotic-loaded implant coatings have primarily investigated the efficacy of prophylactic treatment in periprosthetic models of infection, consisting of rodents (mouse and rat) rabbits, and sheep. Regardless of the drug, antibiotic-coated implants generally showed a 0.3–4.5 log-reduction of colonized bacteria (MSSA or MRSA) in bone and 0.35–4.5 log-reduction on implants in comparison to a non-coated control.^204,207,211,213,217–219^ Diefenbeck et al. demonstrated that plasma chemically oxidized titanium rods coated with gentamicin and gentamicin+tannic acid showed 100% and 90% protection in a rat tibia infection model.^214^ Additionally, Metsemakers et al. demonstrated highly favorable results in a rabbit infection model, where doxycycline-coated intramedullary nails had 100% protection against MSSA (JAR060131; doxycycline-susceptible clinical isolate) and 43% protection against MRSA (LUH15101; doxycycline-resistant clinical isolate).^209^ To date, only one Level 2 randomized-controlled study investigating antibiotic-loaded implant coatings has been performed. In this study, the antibiotic loaded polymer described as Defensive Antibacterial Coating; DAC^®^ was evaluated for its ability to minimize infection in trauma cases.^220^ This 253 patient multi-center European study observed a 4.6% rate of surgical site infections in the uncoated implant control group and zero infections in patients treated with DAC^®^-coated implants. Additionally, no adverse side-effects were observed. The positive outcomes of this clinical study prompt the need for further investigation of the efficacy of antibiotic coated implants.

An essential governing factor in infection management is the drug-release kinetics, which must be assessed in vitro. Ideally a biphasic drug-release profile is warranted, consisting of a bolus drug release to immediately deliver high concentrations of antibiotics to eradicate any bacteria, followed by a sustained drug release above the minimum inhibitory concentration that will kill any remaining bacteria (i.e. bacteria emerging from biofilms). Alternative strategies have also investigated the conjugation of bisphosphonates to conventional antibiotics, such as fluoroquinolones^221–223^ and glycopeptides,^224^ for systemic or local delivery to bone infection sites offering drug depot release kinetics near the bone surface.^225^ However, an alarming issue associated with prophylactic antibiotic implant coatings is the risk of antimicrobial-resistant strains. Antibiotic resistance is estimated to causes 10 million deaths/year by the year 2050.^226,227^ This suggests that significant attention should be directed to the development of novel antimicrobial classes to circumvent the constantly evolving bacterial resistance mechanisms.

In addition to silver and antibiotics, groups have explored the use of antimicrobial peptides (AMPs), bacteriophages (phages), and biofilm dispersal agents^228^ in implant coatings to prevent bacterial attachment and colonization. Various interesting coating strategies have been explored to bind these antimicrobials to implant hardware. Although these topics are beyond the scope of this review, the interested reader is referred to other comprehensive reviews.^229,230^

### Dead space management

As discussed previously, implant-associated osteomyelitis commonly occurs in TJR or trauma cases where the insertion of implants is attributed to the contiguous spread of bacteria and infection. The clinical gold standard for treating implant-associated osteomyelitis is a two-stage surgical revision utilizing PMMA bone cement spacers for the local delivery of antibiotics. Antibiotic-eluting PMMA bone cement spacers are a necessity due to the limited blood flow to the site of infection and the ability to deliver high concentrations of select antibiotics with low systemic exposure.^231^ These spacers remain within the patient for 1–10 weeks and are typically removed in a second procedure, where bone grafting is performed with the installation of new hardware stabilization or external fixation to enable fracture healing.^232–236^ The numerous factors involved in treating infection after fracture fixation, such as the unpredictable nature of bone healing and infection, make each patient a unique case. Despite this unpredictability, reported treatment success rates vary between 70% and 90% as defined by osseous union and infection management.^237–239^ To fully understand the variance in success rates of these procedures, a closer examination of the antibiotic-laden spacers is needed.

The primary advantage of using antibiotic-laden PMMA is the delivery of high concentrations of drug directly to the infection site, which cannot be otherwise achieved by systemic administration due to off-target systemic toxicity. Gentamicin and vancomycin are the most commonly used antibiotics in PMMA spacers for implant-associated osteomyelitis.^240^ These antibiotics are typically chosen because of their broad-spectrum activity, compatibility with polymerization of PMMA, minimal host tissue toxicity, and widespread bioavailability.^241,242^ Despite these characteristics, a universal caveat of antibiotic-laden PMMA spacers is their poor drug release kinetics. In vitro studies demonstrate that only 5%–8% of the total antibiotic incorporated is released and in vivo studies assessing gentamicin release from PMMA also confirm that only 5%–18% of total drug is eluted.^243–249^ Additionally, drug release profiles of antibiotics from PMMA demonstrate an initial burst release within the first 24 h followed by a rapid decrease in release rates.^250,251^ This is detrimental because ideally a cement spacer remains in situ for 6 weeks and the subtherapeutic antibiotic concentrations can lead to microorganism resistance to the incorporated drug.^252,253^ The non-eluting surface of spacers can also be a substrate for bacterial colonization and biofilm formation.^254^ Furthermore, the limited number of antibiotics compatible for incorporation into PMMA are not fully effective against the various microbiological defenses and subpopulations of microorganisms commonly associated with implant-associated osteomyelitis.

These shortcomings of PMMA have set the stage for research efforts to develop an improved vehicle for local drug administration and dead space management. The main design criteria for an improved vehicle are: (1) enhanced drug elution kinetics for a biphasic drug-release profile (i.e. burst release followed by sustained release), and (2) a vehicle that is both biodegradable and osteoconductive. Drug-containing scaffolds that are biodegradable and osteoconductive enable the reduction of the required two-stage revision for PMMA to a single-stage revision, where the drug-containing scaffold not only successfully manages the infection, but also enables bone healing of the defect. Currently, various materials have been successfully loaded with antibiotics for the local delivery of antibiotics clinically, including allograft bone, bioactive glass, calcium sulfates, calcium phosphates, collagen implants, and demineralized bone matrix.^255–261^ Other biomaterials have been developed and investigated in preclinical models ranging from natural polymers, synthetic polymers, ceramics, and composite materials, which have been previously reviewed.^262^

A limiting factor in developing novel biomaterials for the management of dead space in osteomyelitis is the establishment of small animal models with clinical validity. Models utilizing internal fixation plates have only partially been able to partially exchange stabilizing screws with dead space management or complete hardware exchange with no dead space management.^263,264^ Although larger animal models exist, mouse models are preferred due to cost-effectiveness and ability to assess genetic traits for susceptible hosts using genetically modified lines. To date, only one mouse model exists where complete hardware exchange has been performed in a septic critical-sized defect.^265^ In this mouse model of implant-associated osteomyelitis, a mid-diaphyseal infection was established by insertion of a titanium screw inoculated with a bioluminescent strain of MSSA (Xen36).^265^ After 7 days of establishing the infection, examination yielded peri-implant and subcutaneous abscesses, as well as biofilm formation on the screw. At that time, a revision surgery was performed removing the infected screw and debriding any pathological soft tissue. New surgical hardware (polyether ether ketone (PEEK) fixation plate and four screws) were then installed and a 3 mm osteotomy was performed to remove the infected mid-diaphysis of the femur. The resulting defect enabled implantation of an antibiotic-impregnated spacer to assess infection management.

In a follow-up study, 3D-printed calcium phosphate scaffolds (CaPS) with incorporated sitafloxacin and rifampin were implemented into this single-stage revision mouse model of femoral implant-associated osteomyelitis.^266^ A dual coating of PLGA enhanced the mechanical strength and elution kinetics of the scaffolds. Drug-release kinetics exhibited a biphasic release in which an initial burst release was observed within the first 48 h followed by sustained zero-order release, which maintained the local concentration at ~ 900× the minimum inhibitory concentration of each drug. Examination of the concomitant delivery and independent local delivery of sitafloxacin and rifampin from 3D printed CaPS within the mouse infection model revealed improved infection management between the 3D printed CaPS with incorporated sitafloxacin and rifampin and the clinical control, gentamicin-laden PMMA bone cement. Reduced bacterial colonization rates were observed at both 3 and 10 weeks post-revision surgery for the 3Dprinted CaPS. Furthermore, a significant increase in bone formation was observed for 3D printed CaPS with incorporated rifampin in comparison to mice treated with gentamicin-laden bone cement, which underwent an additional revision surgery to insert a 3D printed CaPS with no antibiotics for bone healing.

Collectively, emerging technologies used to create biodegradable and controlled-release antibiotic-laden spacers have demonstrated the ability to improve upon the infection management of the clinical gold standard of antibiotic impregnated PMMA. The additional osteoconductive elements of these spacers enable a reduction of the required two-stage revision to single-stage. Future work must focus on refining the bone-healing potential of these scaffolds by addition of osteoinductive elements to enhance bone regeneration in preparation for definitive large animal studies and clinical trials.

## Conclusion

Osteomyelitis has remained the bane of orthopedic surgery for over a half century, and while there have been many advances in our understanding of the pathophysiological consequences of bone infection during this time, there have been no changes to standard of care treatments. Of note is that while it is well-established that eradication of bacteria from all reservoirs of biofilm is imperative for curative treatment in the clinic, the nature of these bacterial reservoirs in chronic osteomyelitis have been poorly understood. Specifically, the recent discovery of *S. aureus* invasion of the OLCN warrants future experiments to elucidate the mechanism of invasion, and to determine how this privileged environment renders standard of care antibiotic therapies ineffective. Additionally, due to a lack of in vivo studies, the role of intracellular persistence in chronic osteomyelitis remains unclear and warrants further validation studies.

The recent breakthroughs in cancer immunotherapy, most notably checkpoint inhibitors, begs the question if similar advances could be made for osteomyelitis. Now that an important first step has been made by defining the host immune proteome against *S. aureus*, efforts are warranted to translate this new information into diagnostics and vaccines that can better detect ongoing infections, assess response to therapy, and develop adjuvants to antibiotic therapy. In particular, we find that MENSA has great potential to identify the pathogenic organism, distinguish acute versus chronic infection, and assess the patient’s response to therapy.

Finally, the time has come to formally investigate the use of non-FDA-approved antibiotic bone cement, as a means to cure osteomyelitis via bolus high-dose delivery of antimicrobials. In addition to the lack of level 1 clinical evidence to support this practice, it is now known that this foreign body material is rapidly colonized by biofilm bacteria after the initial release of the antibiotics. Moreover, rationally designed, custom 3D-printed antibiotic impregnated spacer technologies have emerged that can achieve both initial high-dose bolus release, and sustained antibiotic release at levels above the MIC. As these technologies may lead to standardized single-stage revisions for bone infection, their clinical investigation is warranted.
